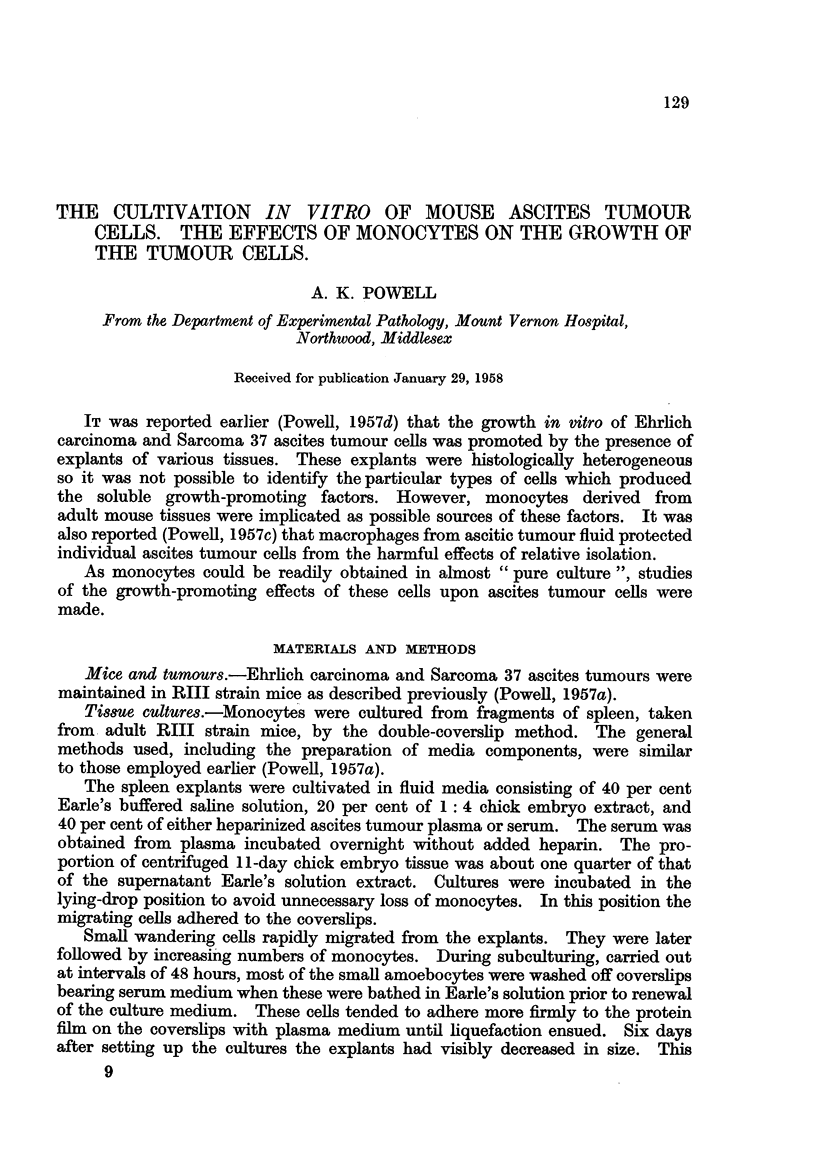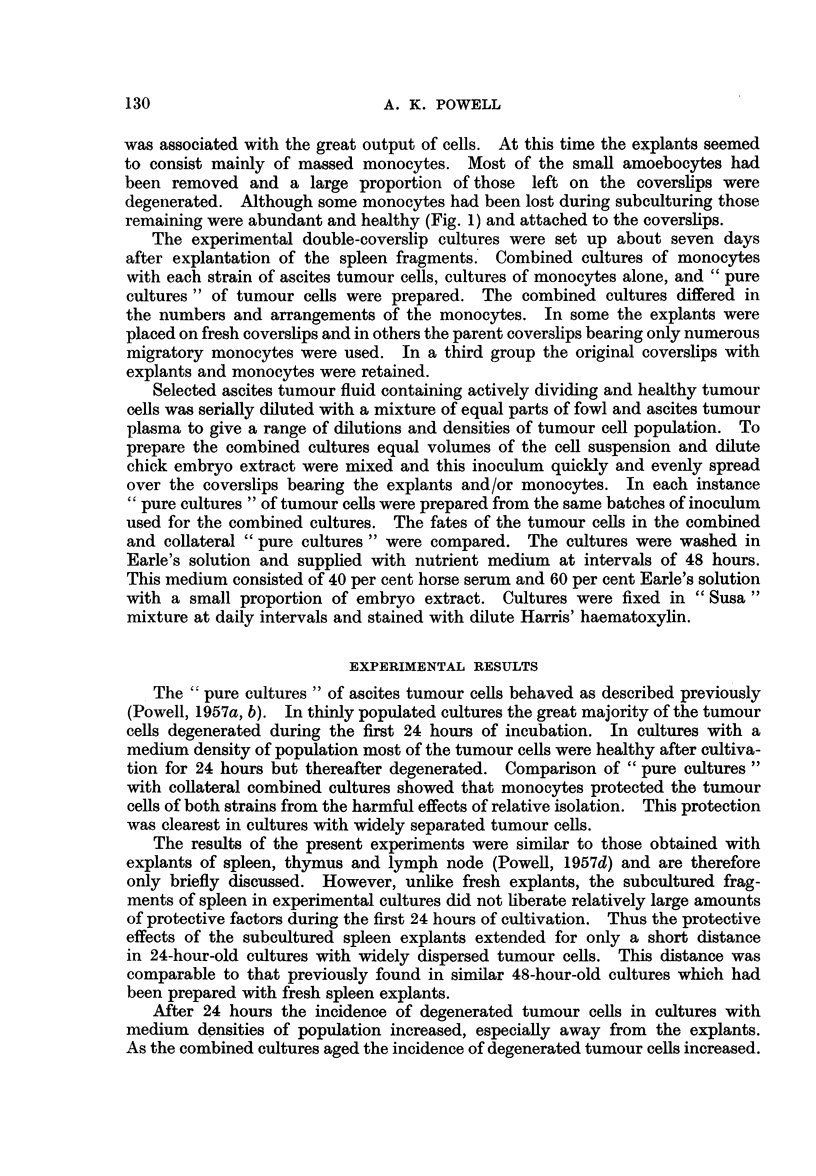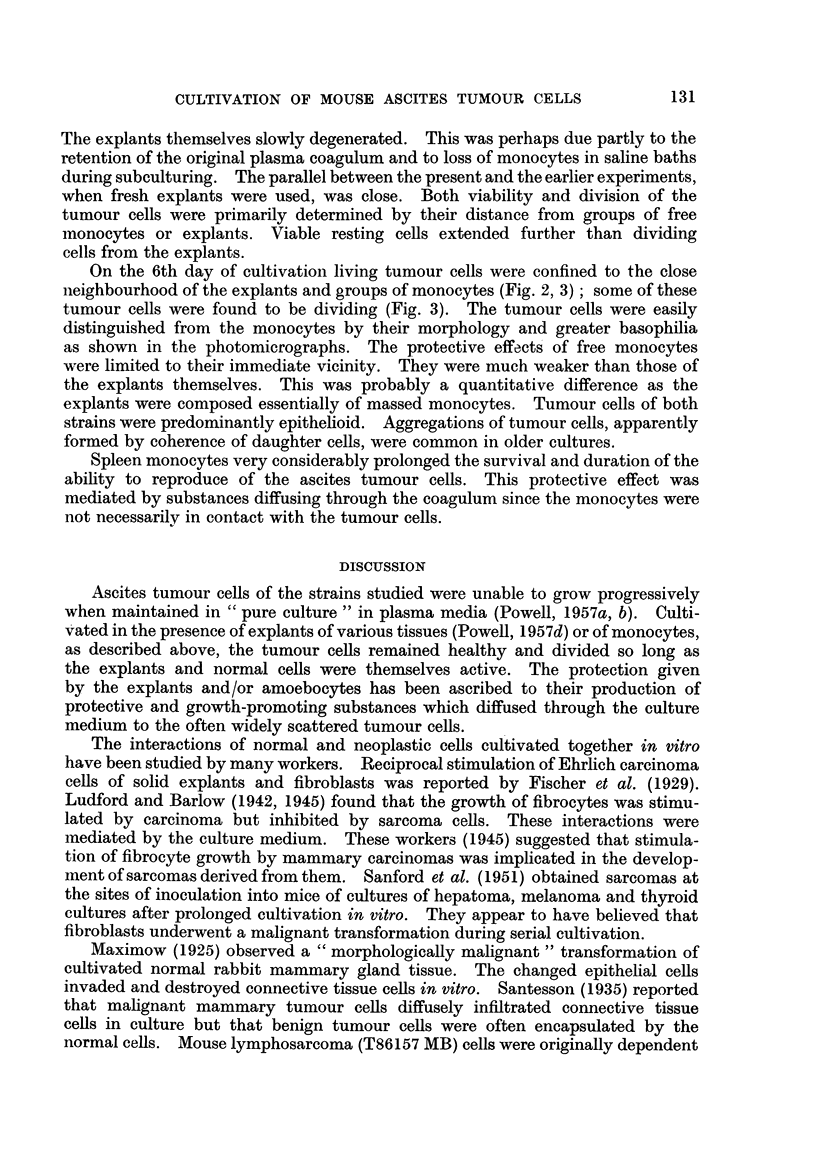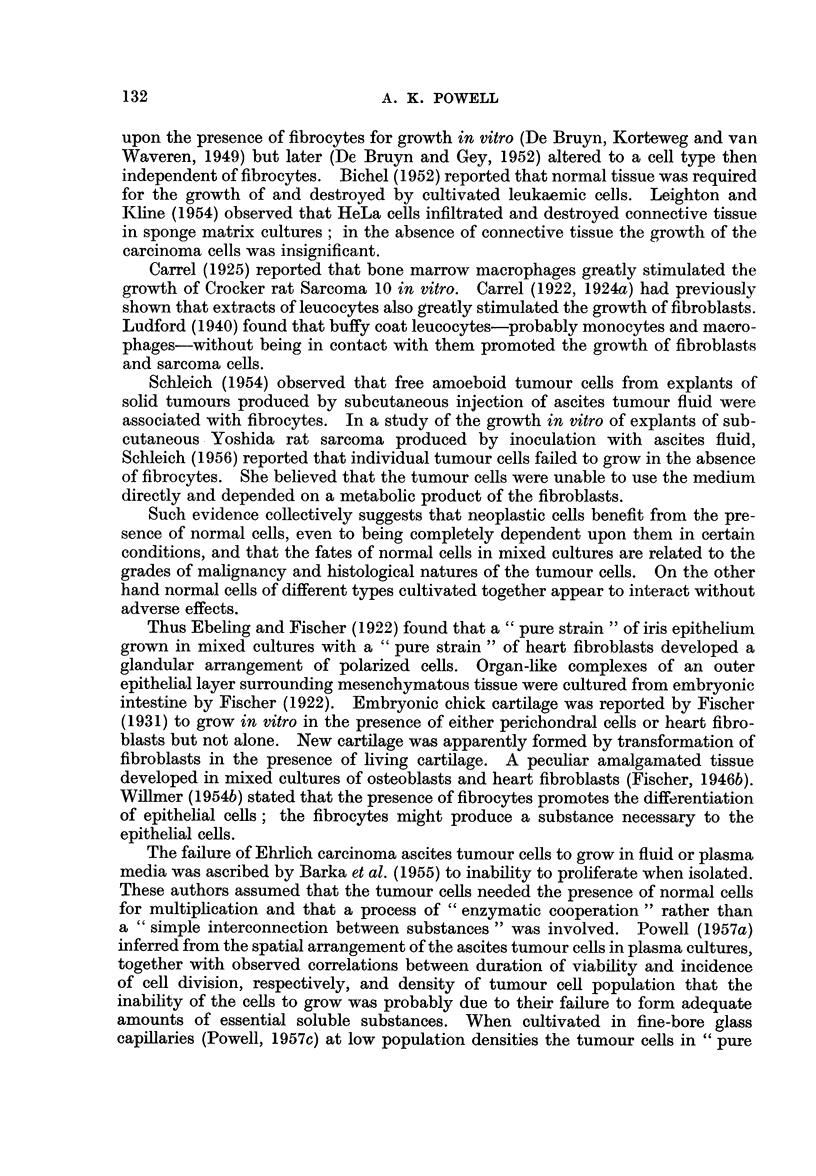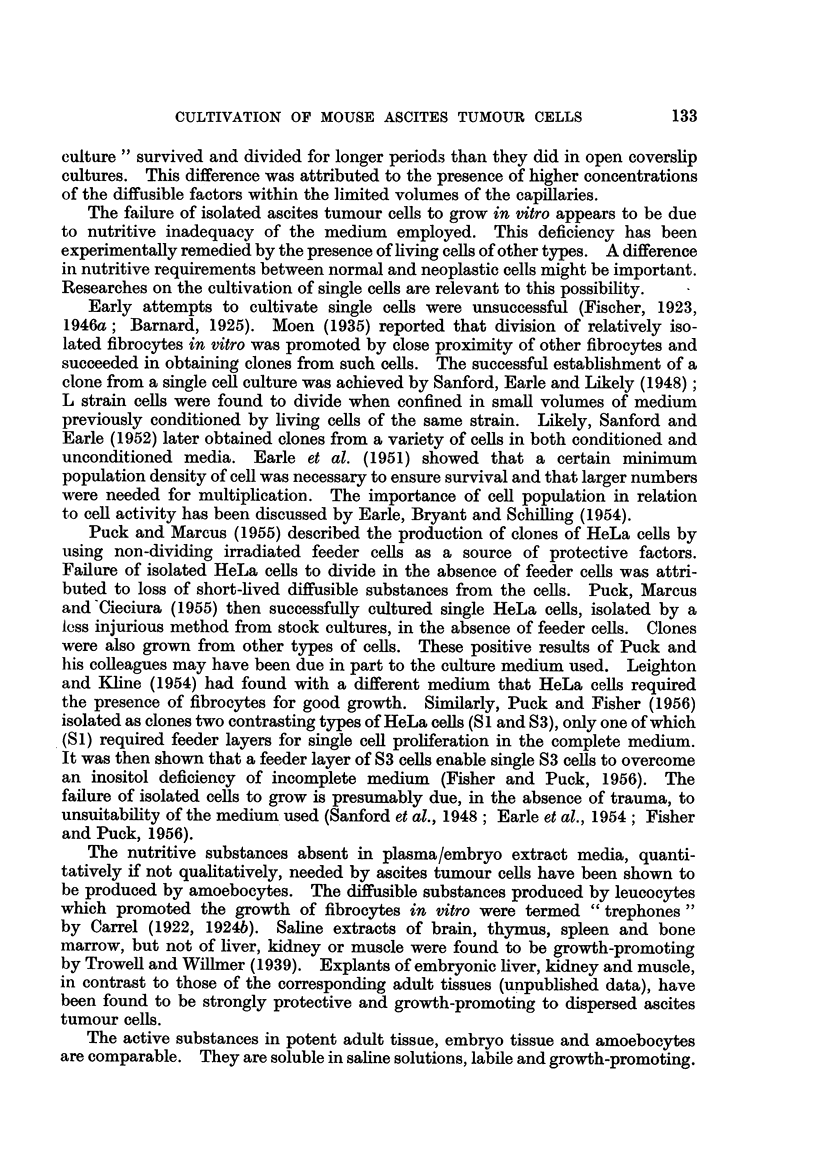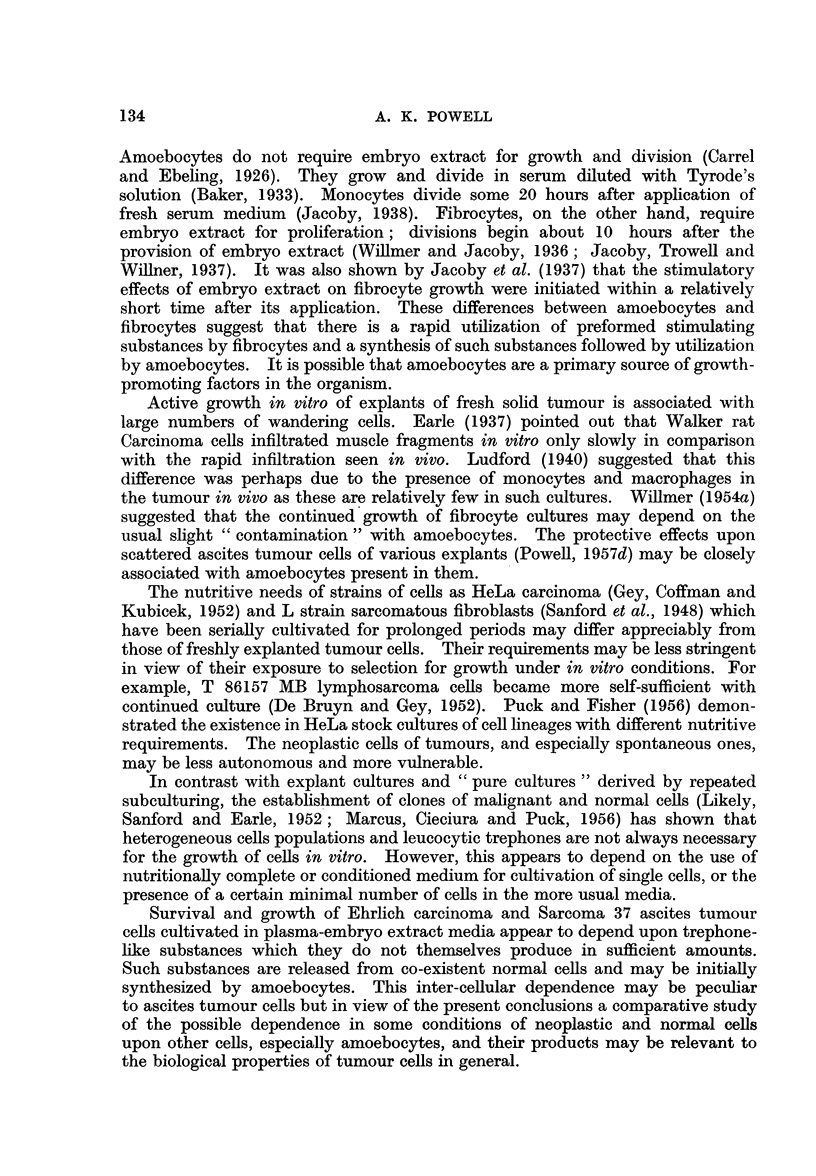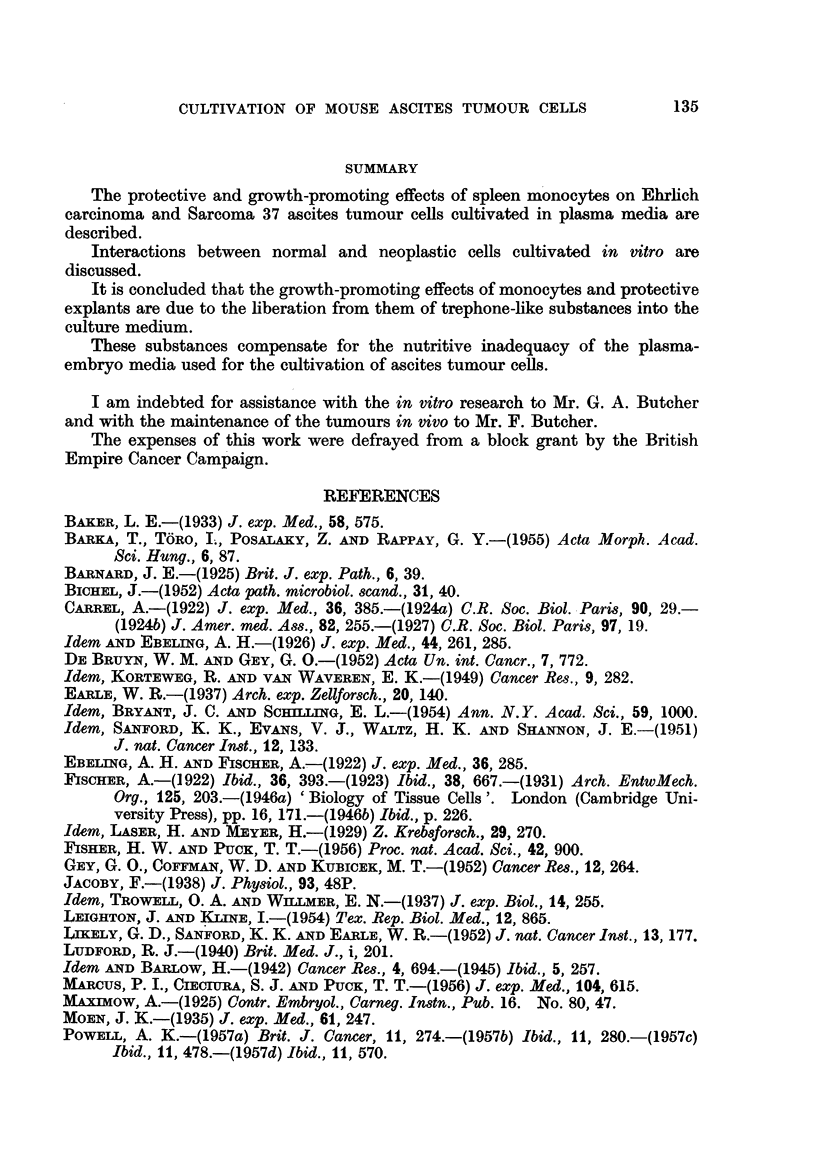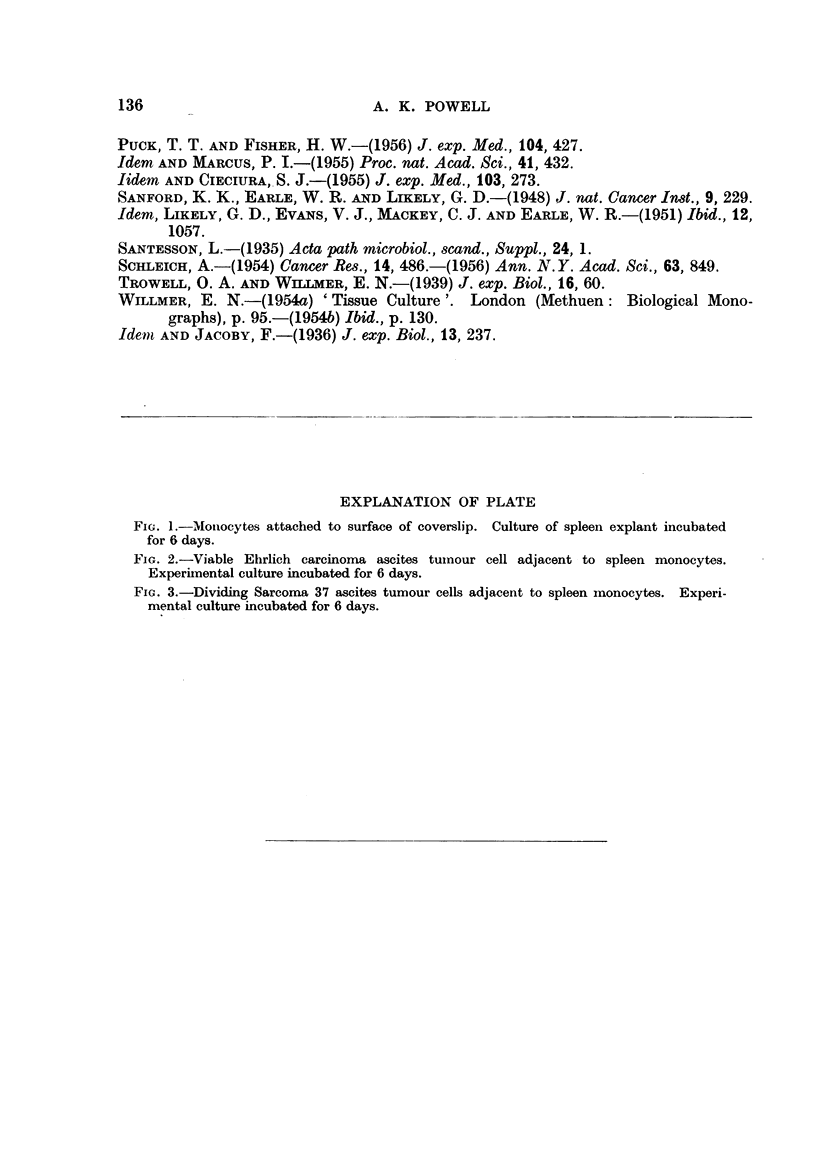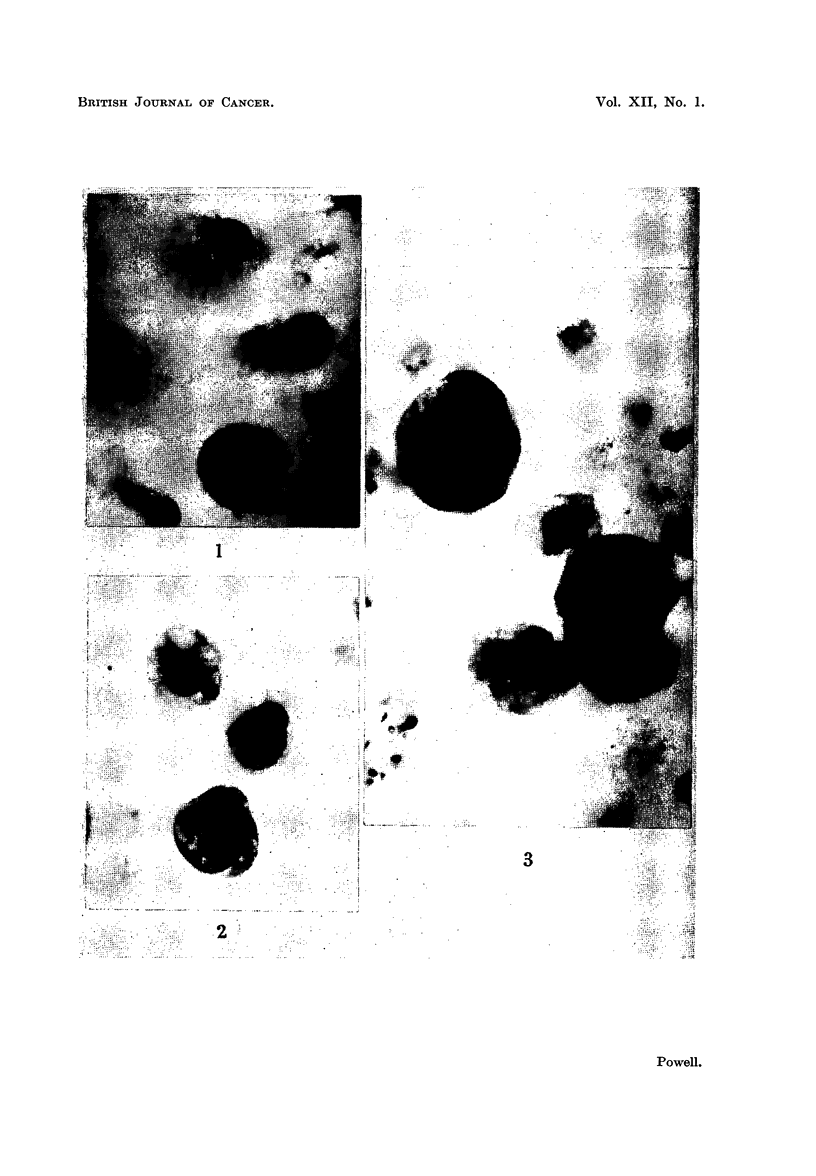# The Cultivation in vitro of Mouse Ascites Tumour Cells. The Effects of Monocytes on the Growth of The Tumour Cells

**DOI:** 10.1038/bjc.1958.17

**Published:** 1958-03

**Authors:** A. K. Powell

## Abstract

**Images:**


### THE CULTIVATION *IN VITRO* OF MOUSE ASCITES TUMOUR CELLS. THE EFFECTS OF MONOCYTES ON THE GROWTH OF THE TUMOUR CELLS.

A. K. POWELL

*From the Department of Experimental Pathology, Mount Vernon Hospital, Northwood, Middlesex*

Received for publication January 29, 1958

It was reported earlier (Powell, 1957*d*) that the growth *in vitro* of Ehrlich carcinoma and Sarcoma 37 ascites tumour cells was promoted by the presence of explants of various tissues. These explants were histologically heterogeneous so it was not possible to identify the particular types of cells which produced the soluble growth-promoting factors. However, monocytes derived from adult mouse tissues were implicated as possible sources of these factors. It was also reported (Powell, 1957*c*) that macrophages from ascitic tumour fluid protected individual ascites tumour cells from the harmful effects of relative isolation.

As monocytes could be readily obtained in almost " pure culture ", studies of the growth-promoting effects of these cells upon ascites tumour cells were made.

### MATERIALS AND METHODS

*Mice and tumours.*—Ehrlich carcinoma and Sarcoma 37 ascites tumours were maintained in RIII strain mice as described previously (Powell, 1957*a*).

*Tissue cultures.*—Monocytes were cultured from fragments of spleen, taken from adult RIII strain mice, by the double-coverslip method. The general methods used, including the preparation of media components, were similar to those employed earlier (Powell, 1957*a*).

The spleen explants were cultivated in fluid media consisting of 40 per cent Earle's buffered saline solution, 20 per cent of 1 : 4 chick embryo extract, and 40 per cent of either heparinized ascites tumour plasma or serum. The serum was obtained from plasma incubated overnight without added heparin. The proportion of centrifuged 11-day chick embryo tissue was about one quarter of that of the supernatant Earle's solution extract. Cultures were incubated in the lying-drop position to avoid unnecessary loss of monocytes. In this position the migrating cells adhered to the coverslips.

Small wandering cells rapidly migrated from the explants. They were later followed by increasing numbers of monocytes. During subculturing, carried out at intervals of 48 hours, most of the small amoebocytes were washed off coverslips bearing serum medium when these were bathed in Earle's solution prior to renewal of the culture medium. These cells tended to adhere more firmly to the protein film on the coverslips with plasma medium until liquefaction ensued. Six days after setting up the cultures the explants had visibly decreased in size. This was associated with the great output of cells. At this time the explants seemed to consist mainly of massed monocytes. Most of the small amoebocytes had been removed and a large proportion of those left on the coverslips were degenerated. Although some monocytes had been lost during subculturing those remaining were abundant and healthy (Fig. 1) and attached to the coverslips.

The experimental double-coverslip cultures were set up about seven days after explantation of the spleen fragments. Combined cultures of monocytes with each strain of ascites tumour cells, cultures of monocytes alone, and " pure cultures " of tumour cells were prepared. The combined cultures differed in the numbers and arrangements of the monocytes. In some the explants were placed on fresh coverslips and in others the parent coverslips bearing only numerous migratory monocytes were used. In a third group the original coverslips with explants and monocytes were retained.

Selected ascites tumour fluid containing actively dividing and healthy tumour cells was serially diluted with a mixture of equal parts of fowl and ascites tumour plasma to give a range of dilutions and densities of tumour cell population. To prepare the combined cultures equal volumes of the cell suspension and dilute chick embryo extract were mixed and this inoculum quickly and evenly spread over the coverslips bearing the explants and/or monocytes. In each instance " pure cultures " of tumour cells were prepared from the same batches of inoculum used for the combined cultures. The fates of the tumour cells in the combined and collateral " pure cultures " were compared. The cultures were washed in Earle's solution and supplied with nutrient medium at intervals of 48 hours. This medium consisted of 40 per cent horse serum and 60 per cent Earle's solution with a small proportion of embryo extract. Cultures were fixed in " Susa " mixture at daily intervals and stained with dilute Harris' haematoxylin.

### EXPERIMENTAL RESULTS

The " pure cultures " of ascites tumour cells behaved as described previously (Powell, 1957*a*, *b*). In thinly populated cultures the great majority of the tumour cells degenerated during the first 24 hours of incubation. In cultures with a medium density of population most of the tumour cells were healthy after cultivation for 24 hours but thereafter degenerated. Comparison of " pure cultures " with collateral combined cultures showed that monocytes protected the tumour cells of both strains from the harmful effects of relative isolation. This protection was clearest in cultures with widely separated tumour cells.

The results of the present experiments were similar to those obtained with explants of spleen, thymus and lymph node (Powell, 1957*d*) and are therefore only briefly discussed. However, unlike fresh explants, the subcultured fragments of spleen in experimental cultures did not liberate relatively large amounts of protective factors during the first 24 hours of cultivation. Thus the protective effects of the subcultured spleen explants extended for only a short distance in 24-hour-old cultures with widely dispersed tumour cells. This distance was comparable to that previously found in similar 48-hour-old cultures which had been prepared with fresh spleen explants.

After 24 hours the incidence of degenerated tumour cells in cultures with medium densities of population increased, especially away from the explants. As the combined cultures aged the incidence of degenerated tumour cells increased.

The explants themselves slowly degenerated. This was perhaps due partly to the retention of the original plasma coagulum and to loss of monocytes in saline baths during subculturing. The parallel between the present and the earlier experiments, when fresh explants were used, was close. Both viability and division of the tumour cells were primarily determined by their distance from groups of free monocytes or explants. Viable resting cells extended further than dividing cells from the explants.

On the 6th day of cultivation living tumour cells were confined to the close neighbourhood of the explants and groups of monocytes (Fig. 2, 3) ; some of these tumour cells were found to be dividing (Fig. 3). The tumour cells were easily distinguished from the monocytes by their morphology and greater basophilia as shown in the photomicrographs. The protective effects of free monocytes were limited to their immediate vicinity. They were much weaker than those of the explants themselves. This was probably a quantitative difference as the explants were composed essentially of massed monocytes. Tumour cells of both strains were predominantly epithelioid. Aggregations of tumour cells, apparently formed by coherence of daughter cells, were common in older cultures.

Spleen monocytes very considerably prolonged the survival and duration of the ability to reproduce of the ascites tumour cells. This protective effect was mediated by substances diffusing through the coagulum since the monocytes were not necessarily in contact with the tumour cells.

### DISCUSSION

Ascites tumour cells of the strains studied were unable to grow progressively when maintained in " pure culture " in plasma media (Powell, 1957*a*, *b*). Cultivated in the presence of explants of various tissues (Powell, 1957*d*) or of monocytes, as described above, the tumour cells remained healthy and divided so long as the explants and normal cells were themselves active. The protection given by the explants and/or amoebocytes has been ascribed to their production of protective and growth-promoting substances which diffused through the culture medium to the often widely scattered tumour cells.

The interactions of normal and neoplastic cells cultivated together *in vitro* have been studied by many workers. Reciprocal stimulation of Ehrlich carcinoma cells of solid explants and fibroblasts was reported by Fischer *et al.* (1929). Ludford and Barlow (1942, 1945) found that the growth of fibrocytes was stimulated by carcinoma but inhibited by sarcoma cells. These interactions were mediated by the culture medium. These workers (1945) suggested that stimulation of fibrocyte growth by mammary carcinomas was implicated in the development of sarcomas derived from them. Sanford *et al.* (1951) obtained sarcomas at the sites of inoculation into mice of cultures of hepatoma, melanoma and thyroid cultures after prolonged cultivation *in vitro*. They appear to have believed that fibroblasts underwent a malignant transformation during serial cultivation.

Maximow (1925) observed a " morphologically malignant " transformation of cultivated normal rabbit mammary gland tissue. The changed epithelial cells invaded and destroyed connective tissue cells *in vitro*. Santesson (1935) reported that malignant mammary tumour cells diffusely infiltrated connective tissue cells in culture but that benign tumour cells were often encapsulated by the normal cells. Mouse lymphosarcoma (T86157 MB) cells were originally dependent upon the presence of fibrocytes for growth *in vitro* (De Bruyn, Korteweg and van Waveren, 1949) but later (De Bruyn and Gey, 1952) altered to a cell type then independent of fibrocytes. Bichel (1952) reported that normal tissue was required for the growth of and destroyed by cultivated leukaemic cells. Leighton and Kline (1954) observed that HeLa cells infiltrated and destroyed connective tissue in sponge matrix cultures ; in the absence of connective tissue the growth of the carcinoma cells was insignificant.

Carrel (1925) reported that bone marrow macrophages greatly stimulated the growth of Crocker rat Sarcoma 10 *in vitro*. Carrel (1922, 1924*a*) had previously shown that extracts of leucocytes also greatly stimulated the growth of fibroblasts. Ludford (1940) found that buffy coat leucocytes—probably monocytes and macrophages—without being in contact with them promoted the growth of fibroblasts and sarcoma cells.

Schleich (1954) observed that free amoeboid tumour cells from explants of solid tumours produced by subcutaneous injection of ascites tumour fluid were associated with fibrocytes. In a study of the growth *in vitro* of explants of subcutaneous Yoshida rat sarcoma produced by inoculation with ascites fluid, Schleich (1956) reported that individual tumour cells failed to grow in the absence of fibrocytes. She believed that the tumour cells were unable to use the medium directly and depended on a metabolic product of the fibroblasts.

Such evidence collectively suggests that neoplastic cells benefit from the presence of normal cells, even to being completely dependent upon them in certain conditions, and that the fates of normal cells in mixed cultures are related to the grades of malignancy and histological natures of the tumour cells. On the other hand normal cells of different types cultivated together appear to interact without adverse effects.

Thus Ebeling and Fischer (1922) found that a " pure strain " of iris epithelium grown in mixed cultures with a " pure strain " of heart fibroblasts developed a glandular arrangement of polarized cells. Organ-like complexes of an outer epithelial layer surrounding mesenchymatous tissue were cultured from embryonic intestine by Fischer (1922). Embryonic chick cartilage was reported by Fischer (1931) to grow *in vitro* in the presence of either perichondral cells or heart fibroblasts but not alone. New cartilage was apparently formed by transformation of fibroblasts in the presence of living cartilage. A peculiar amalgamated tissue developed in mixed cultures of osteoblasts and heart fibroblasts (Fischer, 1946*b*). Willmer (1954*b*) stated that the presence of fibrocytes promotes the differentiation of epithelial cells ; the fibrocytes might produce a substance necessary to the epithelial cells.

The failure of Ehrlich carcinoma ascites tumour cells to grow in fluid or plasma media was ascribed by Barka *et al.* (1955) to inability to proliferate when isolated. These authors assumed that the tumour cells needed the presence of normal cells for multiplication and that a process of " enzymatic cooperation " rather than a " simple interconnection between substances " was involved. Powell (1957*a*) inferred from the spatial arrangement of the ascites tumour cells in plasma cultures, together with observed correlations between duration of viability and incidence of cell division, respectively, and density of tumour cell population that the inability of the cells to grow was probably due to their failure to form adequate amounts of essential soluble substances. When cultivated in fine-bore glass capillaries (Powell, 1957*c*) at low population densities the tumour cells in " pure culture " survived and divided for longer periods than they did in open coverslip cultures. This difference was attributed to the presence of higher concentrations of the diffusible factors within the limited volumes of the capillaries.

The failure of isolated ascites tumour cells to grow *in vitro* appears to be due to nutritive inadequacy of the medium employed. This deficiency has been experimentally remedied by the presence of living cells of other types. A difference in nutritive requirements between normal and neoplastic cells might be important. Researches on the cultivation of single cells are relevant to this possibility.

Early attempts to cultivate single cells were unsuccessful (Fischer, 1923, 1946*a*; Barnard, 1925). Moen (1935) reported that division of relatively isolated fibrocytes *in vitro* was promoted by close proximity of other fibrocytes and succeeded in obtaining clones from such cells. The successful establishment of a clone from a single cell culture was achieved by Sanford, Earle and Likely (1948); L strain cells were found to divide when confined in small volumes of medium previously conditioned by living cells of the same strain. Likely, Sanford and Earle (1952) later obtained clones from a variety of cells in both conditioned and unconditioned media. Earle *et al.* (1951) showed that a certain minimum population density of cell was necessary to ensure survival and that larger numbers were needed for multiplication. The importance of cell population in relation to cell activity has been discussed by Earle, Bryant and Schilling (1954).

Puck and Marcus (1955) described the production of clones of HeLa cells by using non-dividing irradiated feeder cells as a source of protective factors. Failure of isolated HeLa cells to divide in the absence of feeder cells was attributed to loss of short-lived diffusible substances from the cells. Puck, Marcus and Cieciura (1955) then successfully cultured single HeLa cells, isolated by a less injurious method from stock cultures, in the absence of feeder cells. Clones were also grown from other types of cells. These positive results of Puck and his colleagues may have been due in part to the culture medium used. Leighton and Kline (1954) had found with a different medium that HeLa cells required the presence of fibrocytes for good growth. Similarly, Puck and Fisher (1956) isolated as clones two contrasting types of HeLa cells (S1 and S3), only one of which (S1) required feeder layers for single cell proliferation in the complete medium. It was then shown that a feeder layer of S3 cells enable single S3 cells to overcome an inositol deficiency of incomplete medium (Fisher and Puck, 1956). The failure of isolated cells to grow is presumably due, in the absence of trauma, to unsuitability of the medium used (Sanford *et al.*, 1948; Earle *et al.*, 1954; Fisher and Puck, 1956).

The nutritive substances absent in plasma/embryo extract media, quantitatively if not qualitatively, needed by ascites tumour cells have been shown to be produced by amoebocytes. The diffusible substances produced by leucocytes which promoted the growth of fibrocytes *in vitro* were termed " trephones " by Carrel (1922, 1924*b*). Saline extracts of brain, thymus, spleen and bone marrow, but not of liver, kidney or muscle were found to be growth-promoting by Trowell and Willmer (1939). Explants of embryonic liver, kidney and muscle, in contrast to those of the corresponding adult tissues (unpublished data), have been found to be strongly protective and growth-promoting to dispersed ascites tumour cells.

The active substances in potent adult tissue, embryo tissue and amoebocytes are comparable. They are soluble in saline solutions, labile and growth-promoting.

Amoebocytes do not require embryo extract for growth and division (Carrel and Ebeling, 1926). They grow and divide in serum diluted with Tyrode's solution (Baker, 1933). Monocytes divide some 20 hours after application of fresh serum medium (Jacoby, 1938). Fibrocytes, on the other hand, require embryo extract for proliferation; divisions begin about 10 hours after the provision of embryo extract (Willmer and Jacoby, 1936; Jacoby, Trowell and Willner, 1937). It was also shown by Jacoby *et al.* (1937) that the stimulatory effects of embryo extract on fibrocyte growth were initiated within a relatively short time after its application. These differences between amoebocytes and fibrocytes suggest that there is a rapid utilization of preformed stimulating substances by fibrocytes and a synthesis of such substances followed by utilization by amoebocytes. It is possible that amoebocytes are a primary source of growth-promoting factors in the organism.

Active growth *in vitro* of explants of fresh solid tumour is associated with large numbers of wandering cells. Earle (1937) pointed out that Walker rat Carcinoma cells infiltrated muscle fragments *in vitro* only slowly in comparison with the rapid infiltration seen *in vivo*. Ludford (1940) suggested that this difference was perhaps due to the presence of monocytes and macrophages in the tumour *in vivo* as these are relatively few in such cultures. Willmer (1954*a*) suggested that the continued growth of fibrocyte cultures may depend on the usual slight " contamination " with amoebocytes. The protective effects upon scattered ascites tumour cells of various explants (Powell, 1957*d*) may be closely associated with amoebocytes present in them.

The nutritive needs of strains of cells as HeLa carcinoma (Gey, Coffman and Kubicek, 1952) and L strain sarcomatous fibroblasts (Sanford *et al.*, 1948) which have been serially cultivated for prolonged periods may differ appreciably from those of freshly explanted tumour cells. Their requirements may be less stringent in view of their exposure to selection for growth under *in vitro* conditions. For example, T 86157 MB lymphosarcoma cells became more self-sufficient with continued culture (De Bruyn and Gey, 1952). Puck and Fisher (1956) demonstrated the existence in HeLa stock cultures of cell lineages with different nutritive requirements. The neoplastic cells of tumours, and especially spontaneous ones, may be less autonomous and more vulnerable.

In contrast with explant cultures and " pure cultures " derived by repeated subculturing, the establishment of clones of malignant and normal cells (Likely, Sanford and Earle, 1952; Marcus, Cieciura and Puck, 1956) has shown that heterogeneous cells populations and leucocytic trephones are not always necessary for the growth of cells *in vitro*. However, this appears to depend on the use of nutritionally complete or conditioned medium for cultivation of single cells, or the presence of a certain minimal number of cells in the more usual media.

Survival and growth of Ehrlich carcinoma and Sarcoma 37 ascites tumour cells cultivated in plasma-embryo extract media appear to depend upon trephone-like substances which they do not themselves produce in sufficient amounts. Such substances are released from co-existent normal cells and may be initially synthesized by amoebocytes. This inter-cellular dependence may be peculiar to ascites tumour cells but in view of the present conclusions a comparative study of the possible dependence in some conditions of neoplastic and normal cells upon other cells, especially amoebocytes, and their products may be relevant to the biological properties of tumour cells in general.

SUMMARY

The protective and growth-promoting effects of spleen monocytes on Ehrlich carcinoma and Sarcoma 37 ascites tumour cells cultivated in plasma media are described.

Interactions between normal and neoplastic cells cultivated *in vitro* are discussed.

It is concluded that the growth-promoting effects of monocytes and protective explants are due to the liberation from them of trephone-like substances into the culture medium.

These substances compensate for the nutritive inadequacy of the plasma-embryo media used for the cultivation of ascites tumour cells.

I am indebted for assistance with the *in vitro* research to Mr. G. A. Butcher and with the maintenance of the tumours *in vivo* to Mr. F. Butcher.

The expenses of this work were defrayed from a block grant by the British Empire Cancer Campaign.

REFERENCES

Baker, L. E.—(1933) *J. exp. Med.*, **58**, 575.
Barka, T., Töro, I., Posalaky, Z. and Rappay, G. Y.—(1955) *Acta Morph. Acad. Sci. Hung.*, **6**, 87.
Barnard, J. E.—(1925) *Brit. J. exp. Path.*, **6**, 39.
Bichel, J.—(1952) *Acta path. microbiol. scand.*, **31**, 40.
Carrel, A.—(1922) *J. exp. Med.*, **36**, 385.—(1924*a*) *C.R. Soc. Biol. Paris*, **90**, 29.—(1924*b*) *J. Amer. med. Ass.*, **82**, 255.—(1927) *C.R. Soc. Biol. Paris*, **97**, 19.
*Idem* and Ebeling, A. H.—(1926) *J. exp. Med.*, **44**, 261, 285.
De Bruyn, W. M. and Gey, G. O.—(1952) *Acta Un. int. Cancr.*, **7**, 772.
*Idem*, Korteweg, R. and van Waveren, E. K.—(1949) *Cancer Res.*, **9**, 282.
Earle, W. R.—(1937) *Arch. exp. Zellforsch.*, **20**, 140.
*Idem*, Bryant, J. C. and Schilling, E. L.—(1954) *Ann. N.Y. Acad. Sci.*, **59**, 1000.
*Idem*, Sanford, K. K., Evans, V. J., Waltz, H. K. and Shannon, J. E.—(1951) *J. nat. Cancer Inst.*, **12**, 133.
Ebeling, A. H. and Fischer, A.—(1922) *J. exp. Med.*, **36**, 285.
Fischer, A.—(1922) *Ibid.*, **36**, 393.—(1923) *Ibid.*, **38**, 667.—(1931) *Arch. EntwMech. Org.*, **125**, 203.—(1946*a*) ‘ Biology of Tissue Cells ’. London (Cambridge University Press), pp. 16, 171.—(1946*b*) *Ibid.*, p. 226.
*Idem*, Laser, H. and Meyer, H.—(1929) *Z. Krebsforsch.*, **29**, 270.
Fisher, H. W. and Puck, T. T.—(1956) *Proc. nat. Acad. Sci.*, **42**, 900.
Gey, G. O., Coffman, W. D. and Kubicek, M. T.—(1952) *Cancer Res.*, **12**, 264.
Jacoby, F.—(1938) *J. Physiol.*, **93**, 48P.
*Idem*, Trowell, O. A. and Willmer, E. N.—(1937) *J. exp. Biol.*, **14**, 255.
Leighton, J. and Kline, I.—(1954) *Tex. Rep. Biol. Med.*, **12**, 865.
Likely, G. D., Sanford, K. K. and Earle, W. R.—(1952) *J. nat. Cancer Inst.*, **13**, 177.
Ludford, R. J.—(1940) *Brit. Med. J.*, i, 201.
*Idem* and Barlow, H.—(1942) *Cancer Res.*, **4**, 694.—(1945) *Ibid.*, **5**, 257.
Marcus, P. I., Ciecitura, S. J. and Puck, T. T.—(1956) *J. exp. Med.*, **104**, 615.
Maximow, A.—(1925) *Contr. Embryol., Carneg. Instn., Pub.* 16. No. 80, 47.
Moen, J. K.—(1935) *J. exp. Med.*, **61**, 247.
Powell, A. K.—(1957*a*) *Brit. J. Cancer*, **11**, 274.—(1957*b*) *Ibid.*, **11**, 280.—(1957*c*) *Ibid.*, **11**, 478.—(1957*d*) *Ibid.*, **11**, 570.

PUCK, T. T. AND FISHER, H. W.—(1956) *J. exp. Med.*, **104**, 427.
*Idem* AND MARCUS, P. I.—(1955) *Proc. nat. Acad. Sci.*, **41**, 432.
*Iidem* AND CIECIURA, S. J.—(1955) *J. exp. Med.*, **103**, 273.
SANFORD, K. K., EARLE, W. R. AND LIKELY, G. D.—(1948) *J. nat. Cancer Inst.*, **9**, 229.
*Idem*, LIKELY, G. D., EVANS, V. J., MACKEY, C. J. AND EARLE, W. R.—(1951) *Ibid.*, **12**, 1057.
SANTESSON, L.—(1935) *Acta path microbiol., scand., Suppl.*, **24**, 1.
SCHLEICH, A.—(1954) *Cancer Res.*, **14**, 486.—(1956) *Ann. N.Y. Acad. Sci.*, **63**, 849.
TROWELL, O. A. AND WILLMER, E. N.—(1939) *J. exp. Biol.*, **16**, 60.
WILLMER, E. N.—(1954*a*) 'Tissue Culture'. London (Methuen: Biological Monographs), p. 95.—(1954*b*) *Ibid.*, p. 130.
*Idem* AND JACOBY, F.—(1936) *J. exp. Biol.*, **13**, 237.

---

### EXPLANATION OF PLATE

FIG. 1.—Monocytes attached to surface of coverslip. Culture of spleen explant incubated for 6 days.

FIG. 2.—Viable Ehrlich carcinoma ascites tumour cell adjacent to spleen monocytes. Experimental culture incubated for 6 days.

FIG. 3.—Dividing Sarcoma 37 ascites tumour cells adjacent to spleen monocytes. Experimental culture incubated for 6 days.